# Supplementary material for: Visualizing hot carrier dynamics by nonlinear optical spectroscopy at the atomic length scale
Source: Nat Commun. 2025 May 29;16:4999. doi: 10.1038/s41467-025-60384-2 (PMC12122852; doi:10.1038/s41467-025-60384-2)
Supplement: Supplementary file 1 — Supplementary Information [file 41467_2025_60384_MOESM1_ESM.pdf]

Supplementary Information for

Visualizing hot carrier dynamics by nonlinear optical spectroscopy at the atomic length scale

Yang Luo[1,*], Shaoxiang Sheng[1,*], Andrea Schirato[2,3,*], Alberto Martin-Jimenez[1,4], Giuseppe Della Valle[2,5 +], Giulio Cerullo[2,5], Klaus Kern[1,6], Manish Garg[1,+]

[1] Max Planck Institute for Solid State Research, Heisenbergstr. 1, 70569 Stuttgart, Germany

[2] Dipartimento di Fisica, Politecnico di Milano, Piazza L. da Vinci 32, 20133 Milano, Italy

[3] Department of Physics and Astronomy, Rice University, 6100 Main St Houston, Texas 77005, United States

[4] Instituto Madrileño de Estudios Avanzados en Nanociencia (IMDEA Nanociencia), Calle Faraday 9, 28049 Madrid, Spain

[5] Istituto di Fotonica e Nanotecnologie – Consiglio Nazionale delle Ricerche, Piazza L. da Vinci 32, 20133 Milano, Italy

[6] Institut de Physique, Ecole Polytechnique Fédérale de Lausanne, 1015 Lausanne, Switzerland

* These authors contributed equally: Y. Luo, S. Sheng and A. Schirato

+ Authors to whom correspondence should be addressed.

giuseppe.dellavalle@polimi.it and mgarg@fkf.mpg.de

Supplementary Note 1: Experimental Details

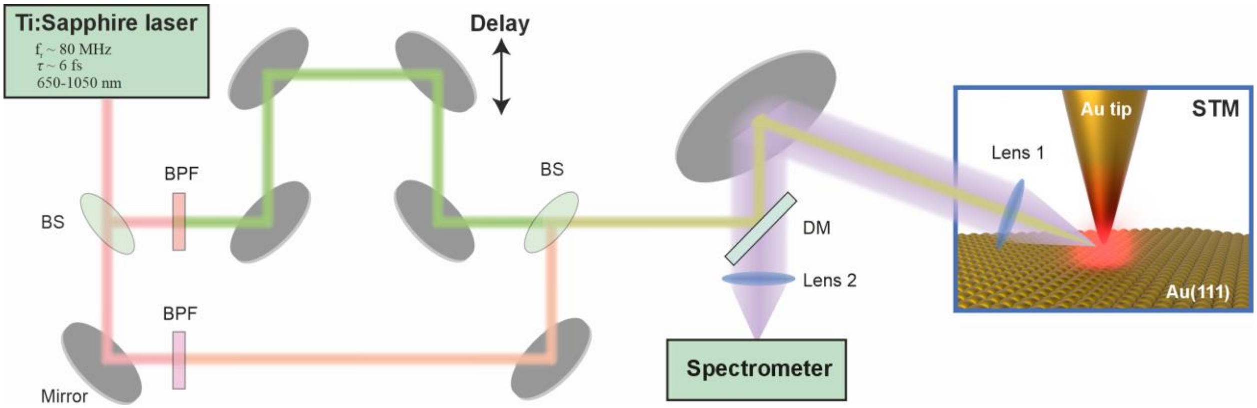

Supplementary Figure 1: Experimental setup. In the two-color pump-probe experiment, pump pulse with the spectral range of ~ 830 – 870 nm (pulse duration ~ 30 fs) and probe pulse with the spectral range of ~ 715 – 750 nm (pulse duration ~ 30 fs) were generated by using different bandpass filters in the two arms of the pump-probe setup. In the single-pulse experiments (Fig. 1, main-text), ultrashort laser pulses with the spectral range of ~715 – 725 nm (pulse duration ~ 80 fs) were generated in one arm, while the other arm of the setup was blocked. BS: beam splitter; BPF: bandpass filter; DM: dichroic mirror.

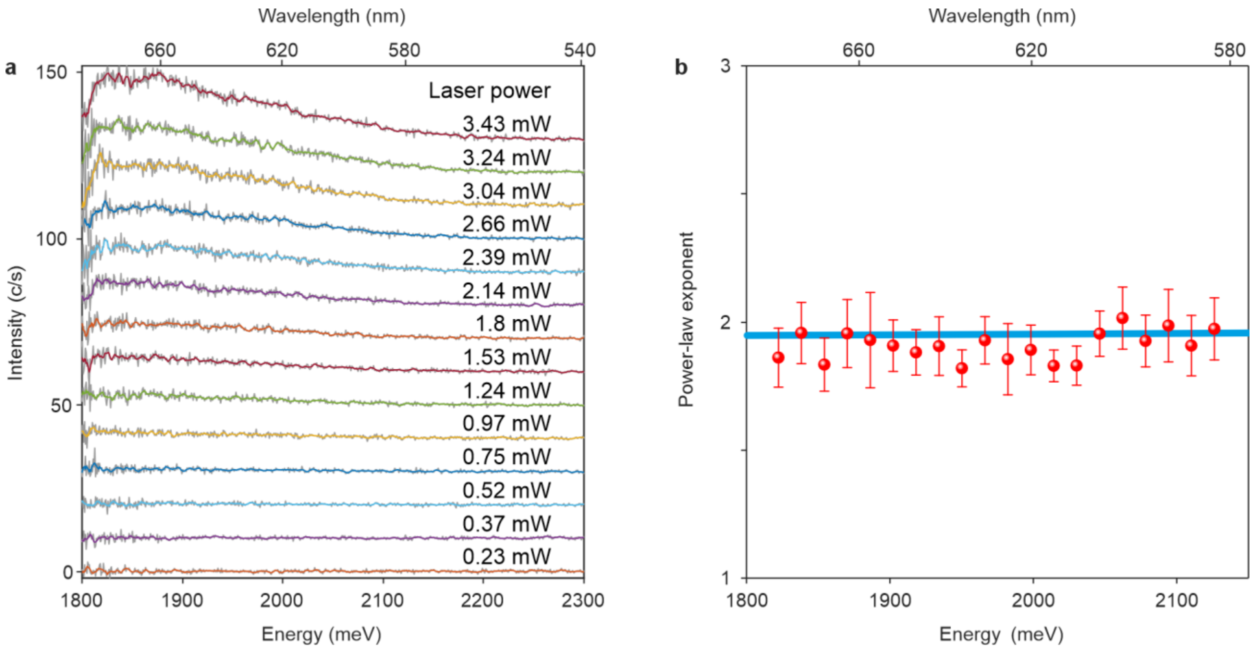

Supplementary Figure 2: Spectrally-resolved power-law exponent in the anti-Stokes spectra. a, A series of anti-Stokes spectra measured at various powers of the incident laser pulses, as annotated on top of each spectrum. Ultrashort laser pulses with the spectral range of ~ 715-725 nm (~ 80 fs) were used to generate the anti-Stokes signal, as indicated in Fig. 1 of the main text. **b**, Energy dependence of the power-law exponent. The spectral intensity at each photon energy (red dots) was obtained by integrating over an energy width of ~16 meV. The error bars indicate the standard deviation from the fit. Top x-axis in **a** and **b** represent the spectral axis in nm. The solid-blue curve represents the spectrally resolved power-law exponent extracted from the simulated anti-Stokes spectra.

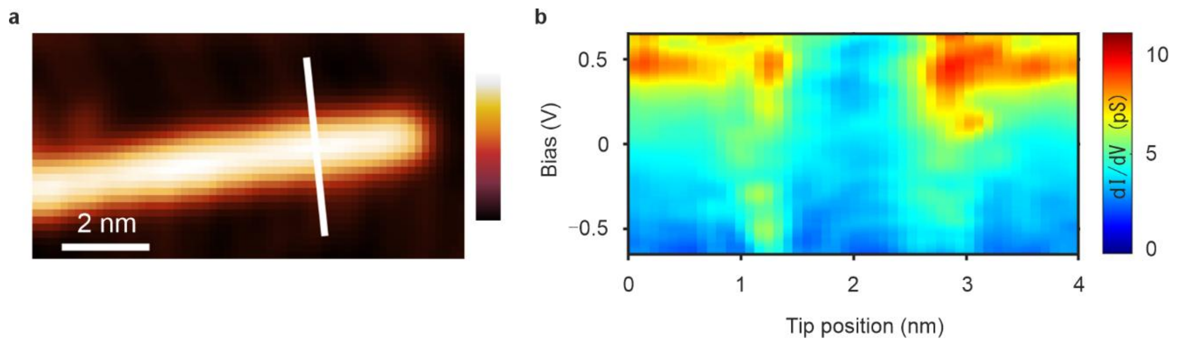

Supplementary Figure 3: Differential conductance measurements on a single GNR. a, STM topography of a single GNR on the Au(111) surface, measured in the constant current mode of the STM junction, with a tunneling current of 33 pA and a bias of 1 V. The color scale represents the relative variation in the tip height during the measurement. **b**, A series of differential conductance (dI/dV) spectra measured along the annotated white line in **a** (from top to bottom). The modulation voltage for the measurement was 40 mV at the frequency of 887 Hz. The height of the nanotip during the measurement (open-feedback) was stabilized at a tunneling current of 100 pA and a bias of 1 V. The color scale represents the conductance. The x-axis represents the tip position, whereas the y-axis denotes the biases used for the differential conductance measurement.

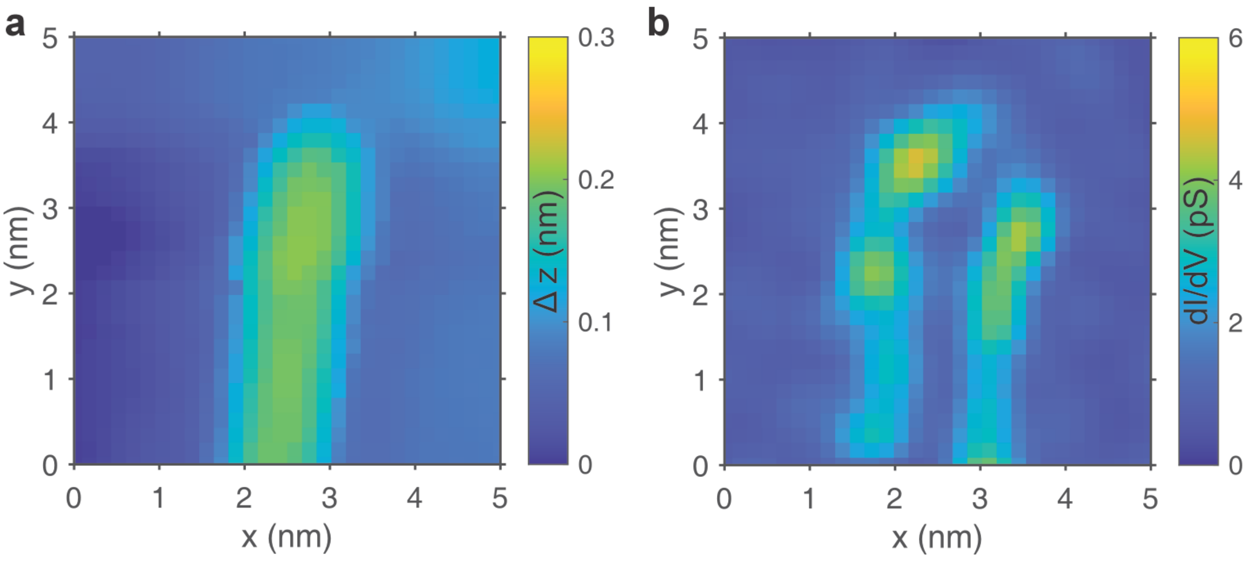

Supplementary Figure 4: Differential conductance mapping of a GNR. a, Constant current STM topographic image of a GNR. The colour bar indicates the variation of the vertical position of the nanotip. **b,** Simultaneously recorded differential conductance map of the GNR at the bias of 1 V. A set point current of 100 pA and a bias of 1 V was used for the STM and STS mapping. The colour bar denotes the value of the differential conductance in pS.

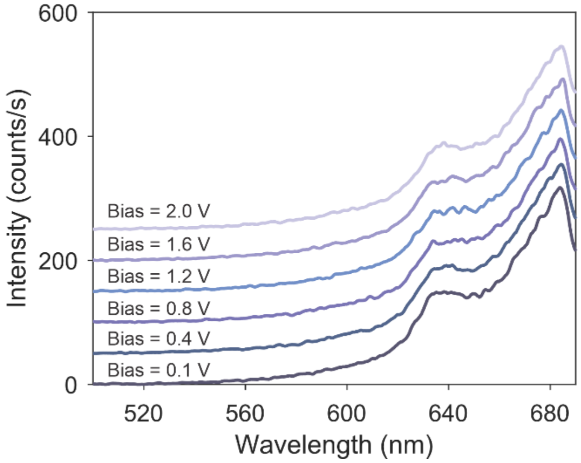

Supplementary Figure 5: Bias dependence of the anti-Stokes spectra. A series of anti-Stokes spectra, measured at zero time delay between the pump and the probe pulses, for various biases applied to the STM junction. The biases used are annotated to each anti-Stokes spectrum. The spectra are vertically shifted for clarity. The STM junction was operated in the constant current mode with the set tunneling current of 1 nA.

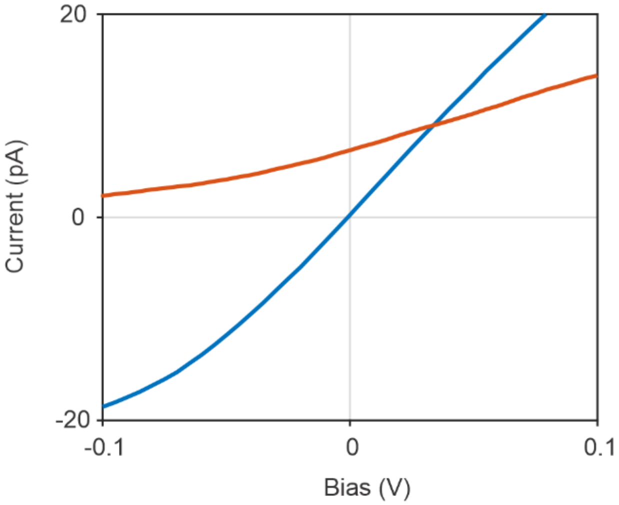

Supplementary Figure 6: Comparison of the variation of the I-V characteristics as a function of the applied bias in the STM junction, in presence (red curve) and absence (blue curve) of illumination with the laser pulses. Substantial laser-induced tunneling current is measured, as visible from a non-negligible current (~ 7 pA) at the zero bias. Tunneling current of 100 pA at the bias of 1V was used before starting the I-V measurement. A single laser pulse of ~ 8 mW power as described in Fig. 1 (main-text) was used for this measurement.

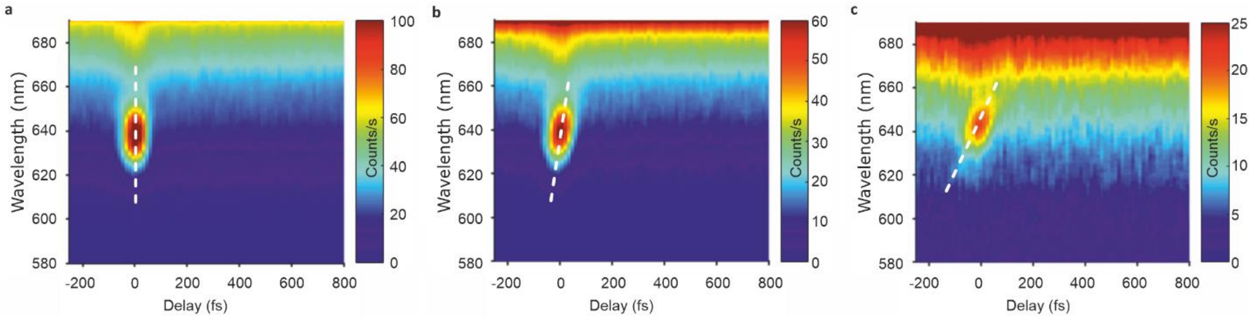

Supplementary Figure 7: Pump-probe measurements in the plasmonic picocavity as a function of the increasing positive chirp of the pump and probe laser pulses. **a**, Chirp-free condition. **b**, Positive chirp by adding 16 mm glass in the pump and probe beam paths. **c**, Positive chirp by adding 32 mm glass in both beam paths. STM junction set point was 8 nA at 100 mV for all the measurements. Vertical white dashed-lines in **a**, **b** and **c** show the increasing positive tilt (chirp) in the FWM signal. The two-color pump-probe experiments in the main text were conducted at the chirp-free condition (**a**).

Supplementary Note 2: Calculation of the local photonic density of states (LPDOS) of the picocavity

To calculate the local photonic density of states (LPDOS) of the plasmonic picocavity under investigation, numerical simulations have been performed using the Finite Element Method (FEM)-based software COMSOL Multiphysics (version 6.2). The system was modelled as a three-dimensional (3D) nanoparticle-on-mirror (NPoM) geometry[1], consisting of a spherical Au nanoparticle (mimicking the STM tip) embedded in a homogeneous environment (here air), and placed at a distance d_{gap} above a semi-infinite flat Au substrate. A schematic of the geometry used in the simulations is shown in Supplementary Figure 8, where d_{gap} = 0.5 nm, akin to the experimental setup. The NP radius was set to 25 nm, to best match the spectral features of the measured LPDOS. However, minor changes of the simulated spectra were obtained by varying the value of the sphere radius, which only slightly modifies the effective longitudinal size of the cavity.

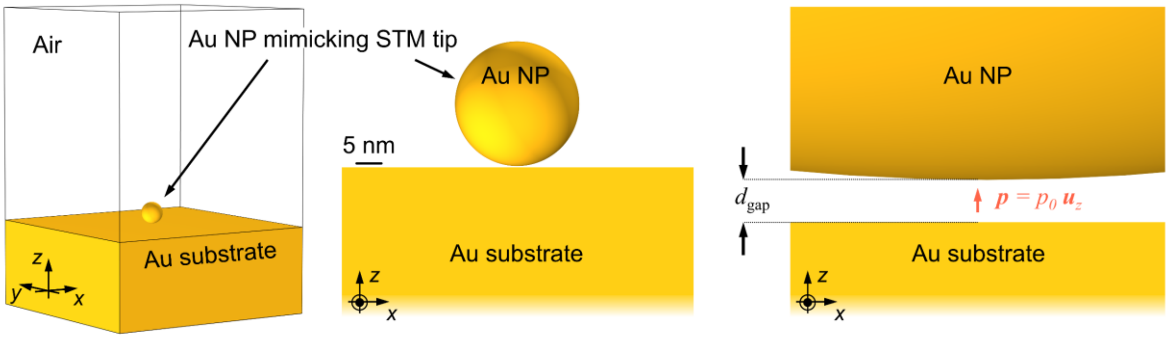

Supplementary Figure 8: Simulations of the plasmonic picocavity LPDOS. Schematic of the NPoM geometry used in the simulations. The zoomed-in sketch of the picocavity (rightmost side) shows the point-like dipolar source (in red, vertically oriented) used to calculate the local photonic density of states.

The LPDOS of the system was then numerically derived from its electromagnetic response upon dipolar excitation, following standard approaches. More precisely, a point-like dipole source with unitary dipole moment was introduced inside the sub-nm gap between the NP and the flat metal surface, and aligned to the vertical (z-) direction, namely expressed as $\boldsymbol{p} = p_0 \cdot \boldsymbol{u_z}$, with p_0 = 1 C·m. In the main simulations, the source was placed at the centre of the cavity, i.e., at $d_{gap}/2$ = 0.25 nm above the metallic surface, and at the same in-plane position as the spherical NP pole. In agreement with previous studies[2], the position of the dipole within the gap in such NPoM configuration only lightly affected the results of the simulations. For the optical properties of Au, the permittivity was described by an analytical expression fitted on experimental data[3], as given in Ref. 4. In addition, to reproduce the broad spectral features in the system optical response, the value of the Drude damping factor Γ_D was increased with respect to the nominal

coefficient for bulk Au[5], Γ_0. A good agreement was found for $\Gamma_D/\Gamma_0 = 1.5$. Maxwell's equations were solved in the full-field formalism, by including perfectly matched layers (PMLs) surrounding the physical domain, and scattering boundary conditions (BCs) beyond the PMLs. To ensure numerical accuracy, the tip region was meshed with particularly fine elements, given a minimum (maximum) size of 0.1 (5) nm. Convergence tests indicated the numerical solution to be stable upon further refinement of the meshing.

Once the electromagnetic field distribution induced by a point-like dipole excitation has been calculated across the whole system, the LPDOS $\rho_{phot}(\mathbf{r}, \omega)$, as a function of the position $\mathbf{r}$ and the dipole source angular frequency ω, can be retrieved according to the following expression[6,7]:

$$\rho_{phot}(\mathbf{r}, \omega) = \frac{4\varepsilon_0}{2\pi\,\omega\,p_0}\mathrm{Re}[\mathbf{E}(\mathbf{r}, \omega)\cdot\boldsymbol{u}_z], \quad \text{(S1)}$$

(in s/m^3) where $\mathbf{r}$ represents the position of the point-like dipolar source.

Note that the expression above provides the total LPDOS, i.e. the sum of its radiative and non-radiative (namely, absorptive) contributions[8]. To distinguish between these two terms, an alternative approach can be employed to compute ρ_{phot} in terms of the system's scattered (i.e., re-radiated) and absorbed powers, P_{rad} and P_{abs}, respectively. Specifically, from the solution of the electromagnetic problem, one can retrieve the following quantities:

$$P_{rad}(\mathbf{r}, \omega) = \int_{\Sigma} \boldsymbol{S} \cdot \mathrm{d}S \quad \text{(S2)}$$

$$P_{abs}(\mathbf{r}, \omega) = \int_{V} Q_{diss}\, \mathrm{d}V \quad \text{(S3)}$$

where $\boldsymbol{S}$ is the Poynting vector, Σ is a surface surrounding the whole system, Q_{diss} represents the absorption Ohmic losses produced within the total volume of metal V (including the spherical NP and the whole Au substrate in our simulations). The two quantities are also considered as functions of the position $\mathbf{r}$ of the dipolar emitter. The corresponding total extincted power P_{tot} is then simply the sum of these two terms, that can be used to express the LPDOS as follows[6]:

$$\rho_{phot}(\mathbf{r}, \omega) = \left[\frac{P_{tot}(\mathbf{r}, \omega)}{P_{vac}(\omega)}\right]\rho_{vac}(\omega) \quad \text{(S4)}$$

where $\rho_{vac}(\omega) = \omega^2/3\pi^2c^3$ is the LPDOS in vacuum, and P_{vac} is the power radiated by a point-like dipole source in vacuum given by Larmor's formula[7]. From the relation given in Eq. S4, it becomes straightforward to identify the two, scattering and absorption, contributions in the total LPDOS, by replacing P_{tot} by either P_{rad} or P_{abs}, respectively. Such distinction is especially relevant since, in agreement with previous reports[9], the meaningful quantity for our analysis is the solely radiative component of the

LPDOS, namely $\rho_{vac}[P_{rad} / P_{vac}]$, as this is the one contributing to the photons detected in our experiments. As such, in all of the expressions and discussions below, the radiative term of the LPDOS will be used. Hereafter, the quantity ρ_{phot} will therefore refer to the radiative LPDOS of the plasmonic picocavity under investigation.

Finally, note that the expressions given above for the LPDOS depend in general on the position of the point-like dipole source defining the excitation. However, in agreement with previous studies treating equivalent NPoM configurations[2], the results of our simulations showed a minor dependence on the position of the dipolar source. For seek of simplicity, we therefore employed directly the LPDOS obtained for a dipole placed at the precise centre of the cavity in the rest of our models.

Supplementary Note 3: Calculation of the far-field optical response of the picocavity

To analyse the interactions between the picocavity and laser light, we modelled the far-field optical behaviour and electromagnetic response of the system upon plane-wave excitation. This allowed us to estimate quantities such as the absorption cross-section and the spatial distribution of the electromagnetic dissipation, relevant to model the photoinduced non-equilibrium carrier dynamics (see Sections below). The simulations were performed on the same 3D geometry discussed above (Supplementary Figure 8), and considering the same optical permittivities of the material. Unlike the calculations detailed in Section II, Maxwell's equations were solved in the scattering formalism, with a background field given by the solution of the electromagnetic problem for a planewave excitation of the structure in the absence of the Au NP (i.e., a semi-infinite metal film with a semi-infinite air layer on top). Such background field was evaluated beforehand numerically, using the COMSOL built-in periodic ports to set the electromagnetic excitation, based on the experimental illumination conditions (sketched in Supplementary Figure 9a). In particular, monochromatic light with a grazing (defined by the wave-vector from the metal film surface) incident angle of 12° was considered, with TM polarisation (in-plane electric field).

Standard formulas[10] were applied to characterise the global optical response of the picocavity. Specifically, we evaluated the absorption, σ_{abs}, and scattering, σ_{sca}, cross-sections of the system, whose simulated spectra are shown in Supplementary Figure 9b. A resonant profile is obtained for both cross-sections, with a peak in scattering at around 680 nm (in agreement with the resonant feature observed in the simulated radiative component of the LPDOS) and a slightly blue-shifted one (at ~660 nm) in absorption, which largely dominates over the former (note the distinct vertical axes).

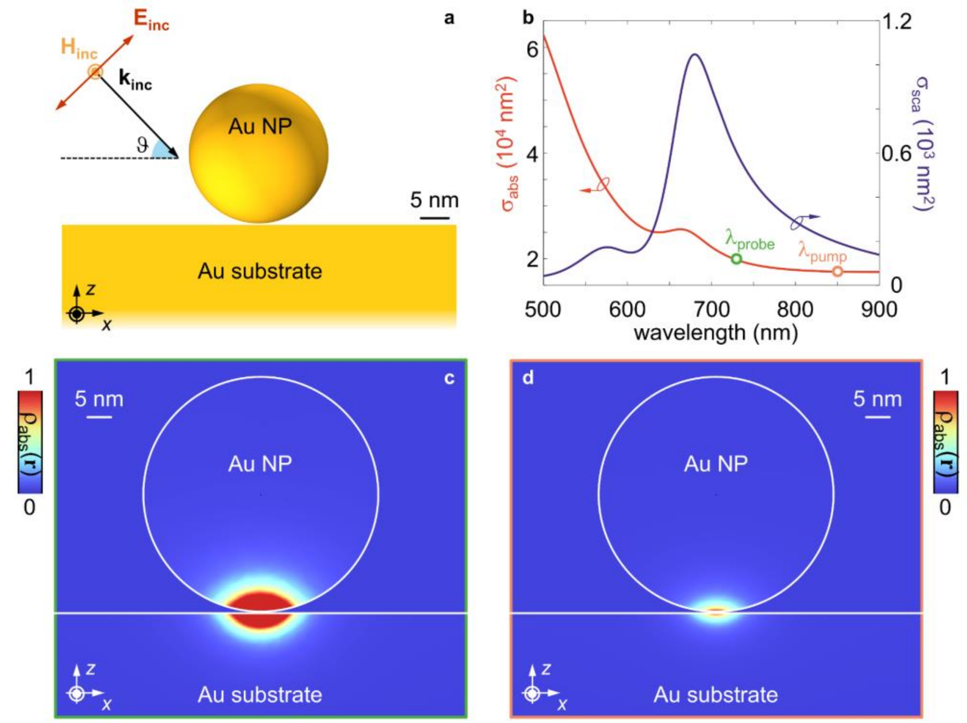

Supplementary Figure 9: Simulations of the plasmonic picocavity optical response. a, Sketch of the numerical geometry and planewave illumination conditions considered in the model. **b,** Simulated spectra of the absorption (red, left vertical axis) and scattering (blue, right vertical axis) cross-sections of the picocavity. **c,** Normalized spatial distribution (two-dimensional cross-cut) of the electromagnetic dissipated power $\rho_{abs}(\mathbf{r})$, evaluated at the probe pulse central wavelength (730 nm). **d,** Same as (c) for the pump pulses central wavelength (850 nm). Both maps in (c) and (d) are normalized to the maximum of the dissipated power at the pump wavelength.

Moreover, the quantitative estimation of the full spectrum of σ_{abs} allowed us to assess consistently the power absorbed by the NPoM system when excited with either pump or probe pulses (their spectral position being highlighted by colored dots in Supplementary Figure 9b). Simulations predict a relatively small (~10%) difference in the σ_{abs} at the central wavelengths of the two pulses (850 nm and 730 nm for pump and probe, respectively), sitting relatively far from the region where the metal losses increase substantially, at shorter wavelengths (< 550 nm). In addition, with the 3D numerical model we could inspect the spatial distribution of the induced fields at the two different wavelengths, providing insight into the volume of the optical modes mediating the interaction. In particular, Figs. S9c and S9d show cross-sectional 2D cuts (in the central *z*-*x* plane, at $y = 0$) of the simulated electromagnetic power density $\rho_{abs}(\mathbf{r}, \lambda)$ (defined as the Ohmic losses inside the metal, and normalised to the maximum of the dissipated power at the pump wavelength) across the system, evaluated at the probe (Supplementary Figure 9c, 730 nm) and pump

(Supplementary Figure 9d, 850 nm) pulses central wavelengths, respectively. The numerical results show the extremely localized character of the optical modes excited at the two wavelengths. The intensity of $\rho_{abs}(\mathbf{r}, \lambda)$ rapidly decays when moving away from the vicinity of the sub-nm cavity gap. Based on these spatial maps, we estimated a typical volume V_{EM} with ellipsoidal shape of semiaxes ~11 nm x 11 nm x 4 nm to approximately describe the spatial extent of the optical modes excited at the frequencies of these pulses. Notably, although these modes are relatively detuned from the scattering peak, they still resemble the field distribution at resonance, which ultimately accounts for their strong confinement and small volumes. This is in fact a distinctive feature of the picocavity architecture. Increasing the gap size not only drastically reduces the field localization, but also significantly alters the field distribution under non-resonant excitation. To illustrate this effect, we performed additional far-field simulations of the picocavity for an increasing distance between the Au substrate and the nanosphere mimicking the experimental STM tip.

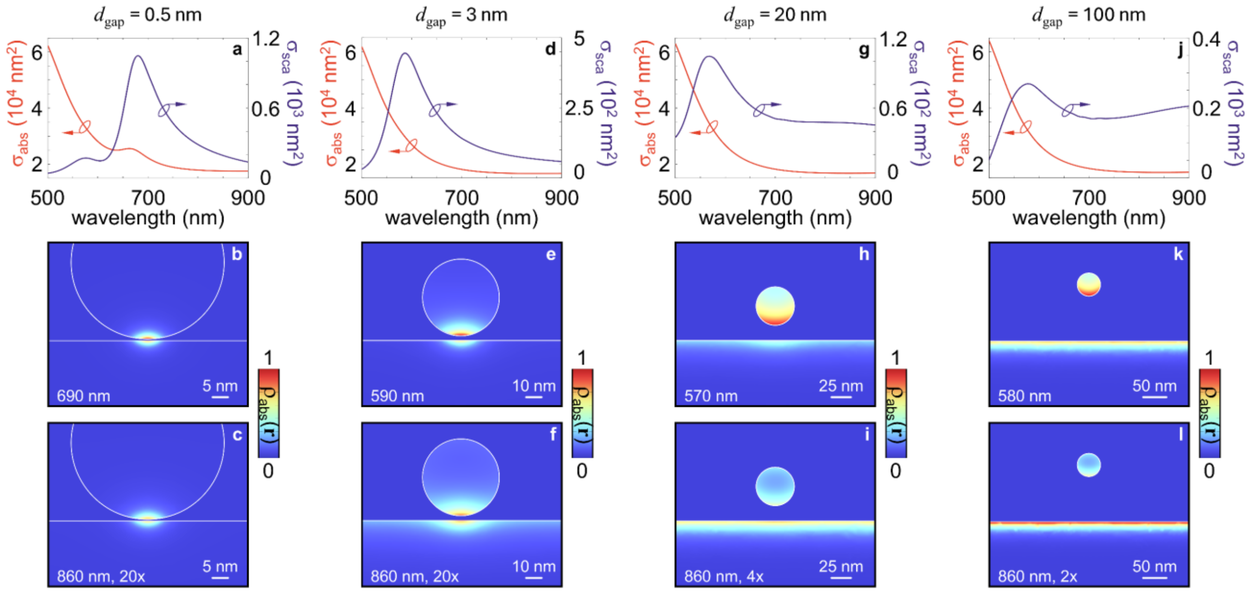

Supplementary Figure 10: Electromagnetic field localisation across the plasmonic picocavity. a, Simulated spectra of the absorption (left-axis) and scattering (right-axis) cross-sections of a picocavity with 0.5 nm gap-size. The same excitation parameters as in Supplementary Figure 9 were considered. **b**, Normalized spatial distribution (cross-sectional 2D cut) of the electromagnetic dissipated power $r_{abs}(r)$, evaluated at the picocavity scattering resonance, 690 nm. **c**, Same as **b** for an non-resonant wavelength of 860 nm. Note the multiplication factor in the spatial map, for the same colour bar as in **b**. **d-f**, Same as **a-c**, for a gap-size of 3 nm. **g-i**, Same as **a-c**, for a gap-size of 20 nm. **j-l**, Same as **a-c**, for a gap-size of 100 nm. The wavelengths (resonant and non-resonant) used in the spatial distribution maps of the electric field are annotated in the respective figures.

Figure S10 shows the main results of our calculations for gap sizes of 0.5 nm (Figs. S10a-S10c), 3 nm (Figs. S10d-S10f), 20 nm (Figs. S10g-S10i) and 100 nm (Figs. S10j-S10l). For each configuration, the absorption and scattering cross-sections are shown (top panels, Figs. S10a, S10d, S10g, S10j), along with the (normalised) dissipation patterns at resonance (middle panels, Figs. S10b, S10e, S10h, S10k) and at a wavelength of 860 nm (bottom panels, Figs. S10c. S10f, S10i, S10l). Numerical results suggest that, for the smallest gap (0.5 nm, Figs, S10a-S10c), the electromagnetic dissipated power evaluated off-resonance is reduced in amplitude (by a factor of ~20) compared to the resonant condition, yet it preserves a spatial distribution reminiscent of the on-resonance case. The field localisation across the picocavity remains strong over a broad bandwidth, and it governs the interaction with light even for wavelengths detuned from the resonance. However, even for a 3 nm gap (Figs. S10d-S10f), while the resonant mode is still confined in the cavity region, a non-resonant excitation exhibits a dissipation pattern that starts to spread over the top surface of the substrate, thus producing a lesser degree of confinement. When the gap size increases further, the cavity mode per se is less and less defined (the nanosphere tends to behave as an isolated scatterer, with its absorption pattern), but, again, an non-resonant excitation produces a dissipation pattern delocalised over the surface of the metallic substrate, in sharp contrast with the 0.5 nm gap-size case.

Supplementary Note 4: Modelling anti-Stokes (aS) emission

The power of the generated anti-Stokes (aS) light was calculated following an approach similar to the one proposed in Refs. 11-13. In essence, given the intensity I of an incoming probe beam, the power of the aS light generated from our picocavity as a function of the frequency ω was expressed as $P_{aS}(\omega)=\sigma_{aS}(\omega)I$, where $\sigma_{aS}(\omega)$ represents the resonant electronic aS Raman scattering cross-section of the picocavity. As for any scattering process, σ_{aS} scales with the square of the (active, i.e. modal) volume V_{EM} of the probe (that we estimated via the electromagnetic simulations presented in Section III), and it is proportional to the local photonic density of states of the picocavity (LPDOS, refer to Section II for the details on how we computed this quantity). Note that in our calculations of the cavity LPDOS, the minor impact of the position of the emitting dipole on the simulated spectra allowed us to treat a space-independent LPDOS, computed for a dipole source located at the centre of the cavity, and here referred to as $\rho_{phot}(\omega)$. Finally, considering the most general situation in our experiments, where a further laser pulse (the pump), was present, σ_{aS} depends also on the pump-probe time delay t. Upon these assumptions, we formulated the following expression for the dynamical aS Raman scattering cross-section:

$$\sigma_{aS}(\omega, t) = A V_{EM}^2 \, \rho_{phot}(\omega) \, J_E(\omega, t), \tag{S5}$$

where $J_E(\omega, t)$ denotes the *joint density of electronic states* that are available to contribute to the aS emission at the considered frequency ω and time delay t, while A is a constant to be determined by comparison with the experiments. More precisely, similarly to the formalism introduced in Ref. 13, we defined $J_E(\omega, t)$ as follows:

$$J_E(\omega, t) = \int_{E_{min}}^{E_{max}} \rho_J(E, E+\hbar\omega, t)\,\mathrm{d}E, \tag{S6}$$

where ρ_J is the energy distribution of the joint density of electronic states between the initial, higher energy, level E_i, and the final, lower energy, level E_f, involved in the emission of an aS photon of energy $\hbar\omega$ (namely such that $E_i = E_f + \hbar\omega$, with $E_f = E$ in Eq. S6 above). The expression for ρ_J reads:

$$\rho_J(E_f, E_i, t) = [f(E_i, t)\ \rho_E(E_i)]\ \{[1 - f(E_f, t)]\ \rho_E(E_f)\}. \tag{S7}$$

In the above formula, $f(E, t)$ is the time-dependent nonequilibrium electron occupancy distribution (see Sections V and VII for details on how we calculated it for the photoexcitation conditions under analysis), and $\rho_E(E)$ is the electronic density of states, that we took from ab-initio calculations previously reported for Au[14]. For the extrema of integration in Eq. S6, we assumed E_{max}= 4.5 eV (from the Fermi level) and $E_{min} = -E_{max}$ thus ensuring to cover with numerical accuracy the entire energy spectrum of the non-equilibrium electronic distribution, featuring occupied states at energies as high as the probe photon energy (~1.7 eV).

With the set of expressions given above, our model provided us with a semi-quantitative estimation of the optical signal measured experimentally. In particular, since no specific assumptions were made regarding the electron energy distribution $f(E)$ in deriving the equations above (Eqs. S5-S7) to express σ_{aS}, the formalism outlined above was applied to describe both single-pulse experiments (Fig. 1 in the main text) and pump-probe measurements (Figs. 2 and 3 in the main text). Moreover, by considering the range of incident photon energies analysed in the experiments (below the Au interband transition threshold[15]), the description we pursued suggested that the measured aS emission can be rationalised as an electronic resonance Raman scattering mechanism.

Supplementary Note 5: Modelling hot carrier ultrafast dynamics

The ultrafast dynamics of high-energy 'hot' carriers resulting from the photoexcitation of the plasmonic picocavity with femtosecond laser pulses has been described numerically by a model adapted from the well-established Three-Temperature Model (3TM)[16]. Generally, the 3TM is a semiclassical rate-equation model for the femtosecond to picosecond dynamics of out-of-equilibrium plasmonic nanostructures[17], detailing the relaxation following ultrafast illumination in terms of three energetic variables: (i) N, the excess energy density stored in a fraction of non-equilibrium carriers referred to as 'nonthermal', as they feature an energy

distribution differing by Δf_{NT} from the equilibrium Fermi-Dirac distribution, characterised by a double-step-like spectral shape involving energies as high as the absorbed photon energy; (ii) Θ_E, the temperature of the 'thermalized' portion of the hot carriers, whose energy distribution differs by Δf_T from the equilibrium Fermi-Dirac distribution at room temperature Θ_0, but can be described in terms of a Fermi-Dirac-like function at a higher temperature Θ_E, i.e. $f(E,\Theta_E) = \left[1+e^{(E-E_F)/k_B\Theta_E}\right]^{-1}$; and (iii) Θ_L, the lattice temperature of the nanostructure. By interlinking N, Θ_E and Θ_L in a set of three coupled ordinary differential equations, the 3TM accounts for the electron-electron and electron-phonon scattering mechanisms towards relaxation, and it thus allows for tracking over time the flow of the excess energy delivered by the incident photons to the metal electrons. To then retrieve the temporal evolution of the non-equilibrium (both nonthermal, NT, and thermal, T) carriers' population in terms of their differential energy occupancy distribution $\Delta f_{NT(T)}$, the following expressions apply, respectively:

$$\Delta f_{NT}(E,\, t) = \delta_{NT}(E)\, N(t), \tag{S8}$$

$$\Delta f_T(E,\, t) = f\,[E,\, \Theta_E(t)] \,-\, f\,[E,\, \Theta_0], \tag{S9}$$

where $\delta_{NT}(E)$ is a double-step-like function[16,18] extending up to the incident photon energy from the Fermi level, and $f(E,\Theta_E)$ is the Fermi-Dirac distribution given above. In passing, note that Eq. S8 treats the energy and temporal dependences as separable, in $\delta_{NT}(E)$ and $N(t)$, respectively, implying that all nonthermal carriers relax at the same rate (following electron-electron scattering events, see below for details). This assumption is foundational to the 3TM, and incorporating an energy-dependent carrier relaxation would require alternative modelling approach[18-22], with increased numerical complexity, hence falling beyond the scope of this work.

For the purpose of modelling the ultrafast response of the picocavity under analysis, given some peculiar features of such plasmonic architecture, we built upon the more conventional 3TM and developed an *ad-hoc* reduced rate-equation model for our system. In particular, dedicated simulations (exhaustively detailed in Section VI) showed that accounting for spatial diffusion of the thermalized hot carriers is critical to accurately model the system's ultrafast dynamics. As more extensively discussed below (refer to Section VI), this peculiar behaviour can be rationalised by considering the extreme localization of the optical modes across the picocavity, and the resulting strong spatial gradients of the electronic excitation. A rigorous description of these mechanisms can be achieved via an inhomogeneous formulation of the 3TM[23,24], where three coupled partial differential equations regulate locally the space-time dynamics of hot carriers, yet at the cost of numerical complexity. Hence, to keep the model handy, we implemented a reduced approach and included the effects of the electronic spatial diffusion by introducing an imbalanced extra de-excitation channel for thermal carriers. The rate of this contribution was defined in terms of an effective diffusion

time $\tau_{\text{diff,eff}}$, and a polynomial in the electronic temperature was employed to mimic the actual diffusion process. Both the value of $\tau_{\text{diff,eff}}$ and the polynomial order were then adjusted to best fit the rigorous solution obtained by solving (only once) the inhomogeneous 3TM (see Section VI). This reduced approach allowed us to drop the explicit space dependence in the model, and resort to a much simpler set of ordinary differential equations in the time-domain simulations.

In formulas, the reduced 3TM we implemented is given by the following set of coupled rate-equations:

$$\frac{\mathrm{d}N}{\mathrm{d}t} = -aN - bN + P_{\text{abs,delay}}(t; t_{\text{d}}), \tag{S10a}$$

$$C_{\text{E}}\frac{\mathrm{d}\Theta_{\text{E}}}{\mathrm{d}t} = aN - G(\Theta_{\text{E}} - \Theta_{\text{L}}) - \frac{1}{\tau_{\text{diff,eff}}}(\Theta_{\text{E}}^{\alpha} - \Theta_{0}^{\alpha}), \tag{S10b}$$

$$C_{\text{L}}\frac{\mathrm{d}\Theta_{\text{L}}}{\mathrm{d}t} = bN + G(\Theta_{\text{E}} - \Theta_{\text{L}}). \tag{S10c}$$

Here, in essence, $a = \left(h\nu_{\text{p}}\right)^2/(2\tau_0 E_{\text{F}}^2)$ is the electron gas heating rate regulating the energy exchange from nonthermal to thermal electrons (with $h\nu_{\text{p}}$ the incident photon energy, E_{F} the Au Fermi energy, and τ_0 a material's constant, here increased to 6 fs relative to estimates from Fermi liquid theory[18] to reflect the lower scattering rates predicted by quantum kinetics calculations[25], a modification that has been shown to accurately match ultrafast pump-probe experiments[26]), b(G) is the scattering rate from nonthermal (thermal) carriers to the phonons in the metal, $C_{\text{E}} = \gamma_{\text{E}}\Theta_{\text{E}}$ (C_{L}) is the electrons (lattice) heat capacity (with γ_{E} a constant), Θ_0 is the temperature of the environment. Given the rather short (< 2 ps) timescales of our analysis, phonon-phonon coupling with the environment was not included in Eq. S10c. Further details on the derivation and values of these parameters can be found elsewhere[17]. Finally, the source term $P_{\text{abs,delay}}(t; t_{\text{d}})$ in Eq. S10a denotes the pulse power density (per unit volume) absorbed by the plasmonic nanostructure, and can be expressed as:

$$P_{\text{abs,delay}}(t; t_{\text{d}}) = \sqrt{4\ln 2/\pi}\,\frac{\sigma_{\text{abs}}(\lambda_{\text{p}})F_{\text{p}}}{V_{\text{EM}}\Delta t_{\text{p}}}\exp\left[-4\ln 2\,(t - t_{\text{d}})^2/\Delta t_{\text{p}}^2\right], \tag{S11}$$

with σ_{abs} and V_{EM} the absorption cross-section and relevant optical mode volume introduced before, F_{p} the pulse fluence, λ_{p} its wavelength, and Δt_{p} its temporal duration (full width at half maximum). Finally, t_{d} is a delay time defining the centre of the Gaussian temporal envelope of the pulse, and acts as a fixed parameter to sweep over to model the pump-probe experiments.

Supplementary Note 6: Spatiotemporal diffusion of hot carriers

Considering the peculiar sub-nm confinement of the picocavity optical modes, the plasmonic hot carriers resulting from photoabsorption inherit a comparable extreme localization within the metal regions in close

proximity to the gap. Strong spatial gradients are thus expected for the electronic excitation, with a relevant role of thermal electrons' ultrafast diffusion away from these intense-field regions. To substantiate this argument and quantify the impact of the electronic space-time evolution, we resorted to a fully inhomogeneous 3TM (I3TM)[23,24] to describe the non-equilibrium dynamics of photoinduced carriers. Note that, in general, also nonthermal hot electrons could experience spatial diffusion[27]. However, we here neglect this effect since it would occur on timescales comparable with their relaxation[23], a process which is accomplished in a few hundreds of femtoseconds (mostly via electron-electron scattering) and expected to dominate their overall dynamics.

In brief, the I3TM extends the formulation of the original 3TM and, while keeping the same thermodynamical approach based on rate equations, it includes the space degree of freedom. Fourier-like terms have been proposed to describe the diffusion of thermal carriers and phonons, and a space-time source (function of **r** and *t*) is considered, based on the specific pattern of light absorption within the nanostructure. In formulas, following previous reports[23,24], the partial differential equations constituting the model read as follows:

$$\frac{\partial N(\mathbf{r},t)}{\partial t} = -\,aN - bN + \rho_{abs}(\mathbf{r})P_{abs,delay}(t;t_d), \tag{S12a}$$

$$C_E\frac{\partial \Theta_E(\mathbf{r},t)}{\partial t} = -\nabla\cdot(-\kappa_E\nabla\Theta_E) - G(\Theta_E - \Theta_L) + aN, \tag{S12b}$$

$$C_L\frac{\partial \Theta_L(\mathbf{r},t)}{\partial t} = \kappa_L\nabla^2\Theta_L + G(\Theta_E - \Theta_L) + bN, \tag{S12c}$$

where the symbols introduced in Section V have the same meaning, κ_E (κ_L) is the electron (lattice) thermal conductivity, and the quantity $\rho_{abs}(\mathbf{r})$, which encodes the space dependence of the drive term, is the (normalized) spatial pattern of the electromagnetic power dissipation density (refer to Section III). More details and the values of the parameters used can be found in previous works.[23,24]

A 3D FEM-based model has been developed using COMSOL Multiphysics (version 6.2) to integrate numerically the I3TM of Eqs. S12a-12c. In the simulations, a simplified geometry (sketched in Supplementary Figure 8) was considered to reduce the computational cost, by leveraging that: (i) the spatial distribution of the electromagnetic absorption $\rho_{abs}(\mathbf{r})$ is strongly confined in a small volume around the gap between the STM tip and metal flat surface, so that regions far from the picocavity do not absorb almost any light, hence do not contribute to the generation of hot carriers; (ii) considering the electronic and the lattice temperatures, both the metal flat surface and the STM tip can be essentially treated as infinite sinks, given their macroscopic spatial extent compared to the nanoscale volume of the cavity mode.

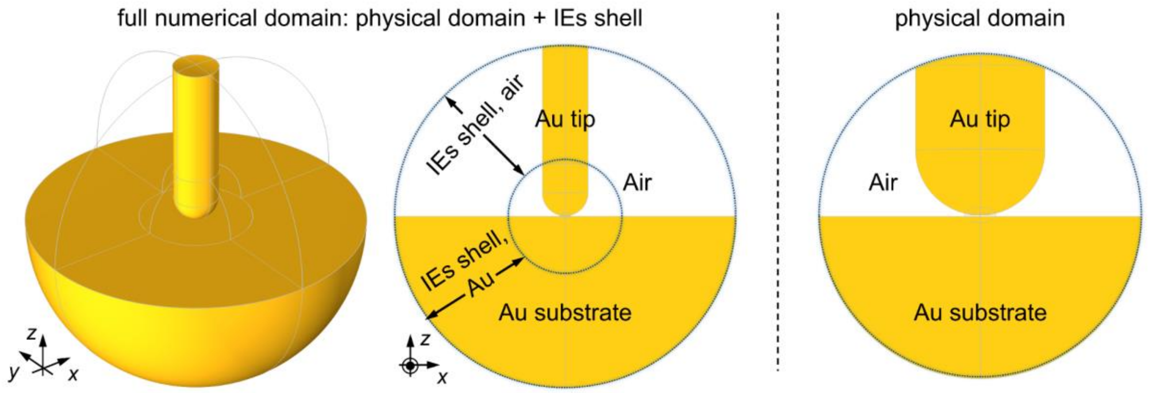

Supplementary Figure 11: Model for the spatiotemporal diffusion of hot carriers. Simplified geometry used to solve numerically the I3TM. 3D (left) and lateral (middle) views of the full numerical domain, including the physical domain and a shell of Infinite Elements (IEs). In the physical domain (right), a sub-nm gap (0.5 nm) between a flat Au surface and a Au tip is considered. The spherical symmetry of the whole domain is chosen to reduce the computational burden of the 3D simulations.

For these reasons, we could reduce the physical domain used in the calculations to a smaller region close to the gap (defined by a radius of ~160 nm), and assign the COMSOL built-in Infinite Elements (IEs) to the surrounding domains, so as to mimic a semi-infinite extent for the air environment, the metallic tip, and the Au substrate. Prior to integrating the I3TM in the time domain, a stationary electromagnetic study was conducted (as detailed in Section III), to numerically estimate $\rho_{abs}(\mathbf{r})$ at the wavelength of the incident pulse and inherit it in Eq. S12a in a fully consistent manner. Finally, to accurately reproduce the experimental conditions, the temperatures initial values were set to $\Theta_E = \Theta_L = \Theta_0 = 11$ K.

As mentioned above (see also Section V), solving the I3TM served us specifically to: (i) corroborate the argument that carriers tend to move away from the proximity of the cavity on ultrafast timescales; and (ii) formulate a homogeneous, reduced rate-equation model (Eqs. S10a-S10c). We therefore tracked the spatiotemporal ultrafast dynamics of thermalized carriers, and the flow of their excess energy content across the metallic regions close to the gap between the tip and the substrate. For that, we monitored the evolution of the electronic temperature increase $\Delta\Theta_E(\mathbf{r},t)$, and the energy density stored in this carrier population fraction, expressed as:

$$\Delta E_E(\mathbf{r},t) = \int_{-\infty}^{t} C_E(\mathbf{r},t') \frac{\partial \Theta_E(\mathbf{r},t')}{\partial t'} \mathrm{d}t'. \tag{S13}$$

In addition, to directly compare the outcome of the I3TM with homogeneous models, these two quantities were averaged over the optical mode volume V_{EM}, that, after inspecting $\rho_{abs}(\mathbf{r})$, we approximated by an

ellipsoid of semiaxes ~11 nm ×11 nm × 4 nm, so as to obtain space-independent relevant quantities. To further facilitate the direct assessment of the carrier diffusion, calculations were performed in the absence of thermal electron-phonon coupling (i.e., $G = 0$ in Eqs. S12b, S12c), so that Fourier-like diffusion was the only available de-excitation channel for Θ_E.

The main results of the simulations are summarised in Supplementary Figure 12, considering an incident pulse of 850 nm wavelength, 30 fs duration, ~0.15 μJ/cm^2 fluence (i.e., typical experimental conditions). In particular, Supplementary Figure 12a shows a spatial map (2D cross-sectional view of the whole 3D geometry) of the electronic temperature field within the metallic regions, evaluated at 50 fs after the pulse peak, namely when Θ_E reaches its peak. The calculations reveal that a maximum temperature increase as low as ~30 K is obtained, which is remarkably low considering that, for the same excitation conditions, a homogeneous 3TM predicts a temperature increase of >500 K. Moreover, $\Delta\Theta_E(\mathbf{r},t)$ exhibits a strongly inhomogeneous spatial distribution, reminiscent of the optical cavity mode regulating the photo-absorption, with the largest values localized around the gap, and a quick drop when moving away from the picocavity region.

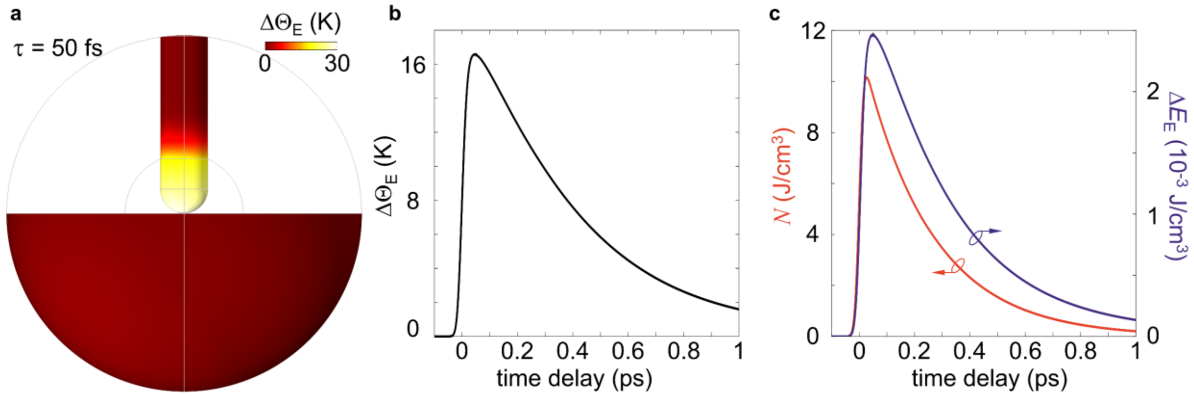

Supplementary Figure 12: Inhomogeneous spatiotemporal diffusion of hot carriers. a, Spatial map (2D cross-sectional view) of the electronic temperature distribution within the metallic (tip and substrate) regions of the picocavity, evaluated at 50 fs after the pulse arrival. **b,** Ultrafast dynamics of Θ_E, averaged over the mode volume. **c,** The ultrafast dynamics of the excess energy stored in the nonthermal portion of electrons N (red, left vertical axis) is compared to that of the thermalized electron energy content ΔE_E (blue, right vertical axis). Both quantities are space-averaged over the cavity mode volume.

Due to such a strong confinement in very small volumes, extreme temperature gradients are obtained, making the diffusion term in Eq. S12b dominate over the carrier ultrafast dynamics. As displayed in Supplementary Figure 12b, $\Delta\Theta_E$ (averaged over the mode volume) undergoes a peculiar temporal evolution, and reaches almost full relaxation within ~1.5 ps, a noticeably short time compared to the standard dynamics of carriers in plasmonic nanostructures, requiring rather tens of ps to go back to equilibrium[17]. The same peculiar dynamics is observed also for ΔE_E, which is reported in Supplementary Figure 12c and compared with the excess energy density stored in the nonthermal fraction of hot carriers, N (both space-averaged over V_{EM}). Unlike any other more conventional plasmonic nanostructure, the two energy contents follow a comparable, sub-ps relaxation, with the contribution of thermal carriers being substantially smaller than the nonthermal ones (note the two distinct vertical axes of the plot, differing by ~3 orders of magnitude).

Finally, as the last step of our modelling approach, we leveraged the accurate solution of the I3TM to approximate the spatiotemporal diffusion of thermal electrons with an effective, more handy term to include in a space-independent rate equation for Θ_E. We assumed this extra de-excitation term to be a polynomial (of order α) in the electronic temperature associated with a characteristic time $\tau_{\mathrm{diff,eff}}$, namely of the form $-\frac{1}{\tau_{\mathrm{diff,eff}}}(\Theta_E^{\alpha} - \Theta_0^{\alpha})$, with Θ_0 the environment temperature (here set to 11 K).

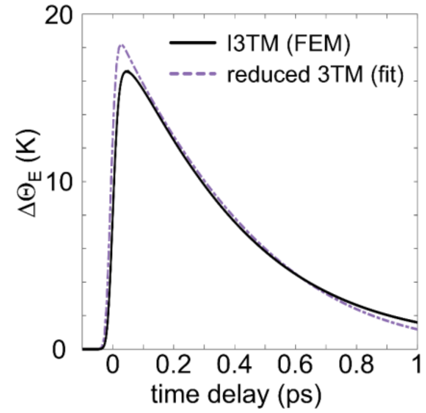

Supplementary Figure 13: Fit of the electronic temperature dynamics. The ultrafast dynamics of the electronic temperature (averaged over the mode volume) obtained by integrating the I3TM in our FEM model (black solid line) is compared with that retrieved from a modified homogeneous 3TM (purple dashed line), where a polynomial of order α with a characteristic time $\tau_{\mathrm{diff,eff}}$ mimics the electron temperature diffusion. The best fit is achieved for $\alpha = 3$ and $\tau_{\mathrm{diff,eff}} = 0.7$ fs.

Both $\tau_{\mathrm{diff,eff}}$ and α were used as fitting parameters. To determine their values, the dynamics of the space-averaged $\Delta\Theta_E$ obtained by integrating the I3TM in COMSOL (Supplementary Figure 12b) was compared

with the numerical solution of the modified 3TM given by Eqs. S10a-S10c above. The best fit (results shown in Supplementary Figure 13) was found for a third-order polynomial ($\alpha = 3$ in Eq. S10b) and an effective diffusion time constant $\tau_{\text{diff,eff}}$ = 0.7 fs. Note that the values obtained for these two parameters of the reduced 3TM should be intended as the result of a fitting procedure. That is, they represent effective quantities introduced to mimic the spatial diffusion of carriers in a simplified formulation of the electron spatiotemporal dynamics, hence do not immediately relate to the mechanisms behind of spatial diffusion. Their values are rather tuned to match the exact results obtained from the I3TM (Eqs. S12a-S12c), where spatial gradients and the electronic thermal conductivity regulate rigorously the evolution of carriers within the metal.

Supplementary Note 7: Modelling the ultrafast modulation of anti-Stokes emission

In order to simulate the pump-driven ultrafast modulation of the probe-induced anti-Stokes (aS) emission, the modelling ingredients outlined above were combined to determine the variations over time of the aS emitted power P_{aS} (see Section IV) due to a non-equilibrium electronic distribution Δf.

More in detail, a nested approach was implemented, according to the following schematic algorithm:

(i) Pump excitation: our modified 3TM (Eqs. S10a-S10c, with the source term given by Eq. S11) was solved for an ultrashort pulse mimicking the interaction with the pump beam, centred around a reference time $t_{\text{d}} = 0$. All the relevant parameters of the simulated pulse (duration, central wavelength, fluence) were set as in the experiments. This calculation provided us with the ultrafast dynamics of the three energetic variables, $N_{\text{pu}}(t)$, $\Theta_{\text{E,pu}}(t)$, and $\Theta_{\text{L,pu}}(t)$, triggered by the pump photons absorption. The simulation was performed over a timescale comprised between $t_{-\infty} = -0.4$ ps and t_{∞}= 1.5 ps.

(ii) Probe excitations: the outcome of the pump pulse simulation was used to set the initial conditions in a series of 3TMs mimicking the interaction with the probe, and iteratively solved by scanning the pump-probe time delay. Practically, we fixed a time delay $t_{\text{d,i}}$ and expressed the source term $P_{\text{abs,delay}}\left(t;t_{\text{d,i}}\right)$ accordingly (Eq. S11), based on the experimental optical parameters of the probe pulse. We then built our 3TM (Eqs. S10a-S10c) for three variables $N_{\text{pr}}(t)$, $\Theta_{\text{E,pr}}(t)$, and $\Theta_{\text{L,pr}}(t)$, whose initial condition was the solution of the pump-excitation model at the considered time delay $t_{\text{d,i}}$, namely $N_{\text{pr}}(t = t_{-\infty}) = N_{\text{pu}}(t = t_{\text{d,i}})$, $\Theta_{\text{E,pr}}(t = t_{-\infty}) = \Theta_{\text{E,pu}}(t = t_{\text{d,i}})$, $\Theta_{\text{L,pr}}(t = t_{-\infty}) = \Theta_{\text{L,pu}}(t = t_{\text{d,i}})$. Integrating the rate equations of the 3TM provided us with the whole temporal evolution (from $t_{-\infty}$ to t_{∞}) of the three probe-quantities, of which we extracted only their value at the delay of interest, namely $N_{\text{pr}}(t = t_{\text{d,i}})$, $\Theta_{\text{E,pr}}(t = t_{\text{d,i}})$, and $\Theta_{\text{L,pr}}(t = t_{\text{d,i}})$. Then, to reconstruct the actual dynamics as a

function of the pump-probe delay, the parameter $t_{d,i}$ was scanned from $t_{-\infty}$ to t_{∞}, and the probe-3TM was iteratively solved for each value of the delay. By collecting the series of solutions at each $t_{d,i}$, we thus retrieved $N_{pr}(t_d)$, $\Theta_{E,pr}(t_d)$, and $\Theta_{L,pr}(t_d)$ over the entire pump-probe delay time window relevant to the experiments.

(iii) Electronic distribution and aS emission rate: we considered, in the most general conditions, the electronic contribution to the aS emission (and in particular, the aforementioned joint density of electronic states $J_E(\omega, t)$, Eq. S6 in Section IV) as the superposition of three terms related to (i) the equilibrium occupancy distribution of electrons, hence time-independent and typically very weak, especially at cryogenic temperature; (ii) the excitation driven by the pump, centred at time delay $t_d = 0$ and expressed by the carrier distribution modulations $\Delta f_{NT,pu}(E,t)$ and $\Delta f_{T,pu}(E,t)$ (refer to Eqs. S8 and S9, respectively), evolving in time according to the dynamics of $N_{pu}(t)$ and $\Theta_{E,pu}(t)$ obtained in the first step above; and (iii) the excitation driven by the probe, depending on the pump-probe time delay and mostly dominating over the others. To compute this term, we employed the dynamics of $N_{pr}(t_d)$ and $\Theta_{E,pr}(t_d)$ obtained from the nested 3TMs in the previous step, and determined the corresponding non-equilibrium distribution $f_{pr}(E,t_d) = f(E, \Theta_0) + \Delta f_{NT,pr}(E,t_d) + \Delta f_{T,pr}(E,t_d)$. Finally, each of these terms was plugged in the (differential with respect to equilibrium) expressions of ρ_J (Eq. S7) and $J_E(\omega, t)$ (Eq. S6) to calculate the aS power P_{aS}.

A comprehensive picture of the typical outcome of our numerical model is presented in Supplementary Figure 14. In particular, Supplementary Figure 14a and 14b show the differential map of the ultrafast aS emission modulation (that is, the same quantity reported in Fig. 3d of the main text), disentangled in its contributions arising from nonthermal and thermalised hot carriers, respectively. Notably, Supplementary Figure 14a closely resembles the total map of Fig. 3 in the main text, and exceeds its thermal carrier counterpart by several orders of magnitude (note the magnification factor in Supplementary Figure 14b). This stark contrast clearly shows that the measured signal is largely dominated by the ultrafast contribution of nonthermal hot carriers.

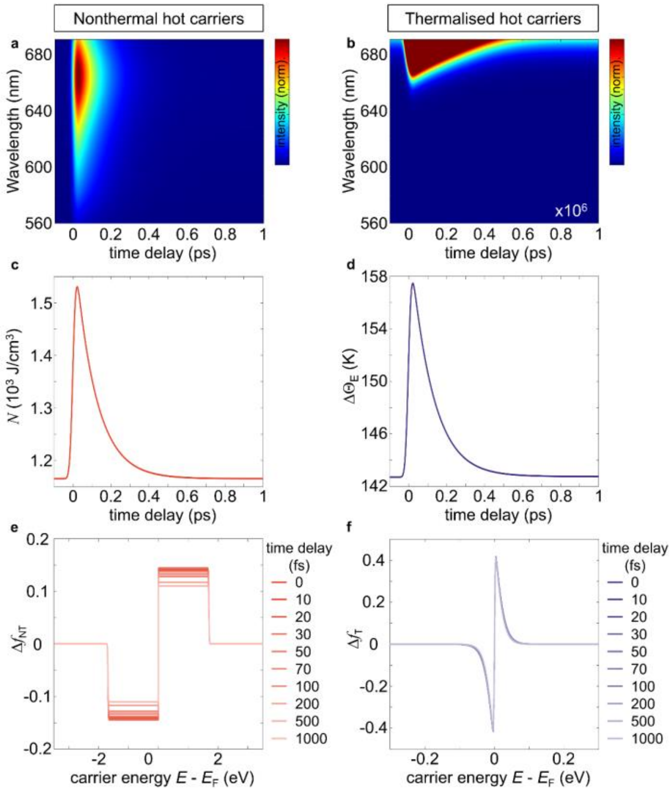

Supplementary Figure 14: Contributions of the nonthermal and thermal carriers in the measured dynamics. a, Disentangled differential map of the ultrafast modulation of the anti-Stokes spectra as a function of the pump-probe time delay, due to nonthermal carriers only. The same excitation conditions as in Fig. 3 of the main text were considered in the simulation. **b,** Same as **a**, disentangling the contribution of the thermal carriers only. Note the magnification factor of the signal amplitude. Both **a** and **b** are normalized to the maximum value in **a**, with a 10^6 multiplication factor in **b**. **c,** Ultrafast dynamics of $N(t)$, the excess energy density stored in the nonthermal fraction of the population of carriers photoexcited by the pump pulse. **d,** Ultrafast dynamics of the corresponding electronic temperature increase, $\Delta\Theta_E(t)$. **e,** Photoinduced variation of the electron energy occupancy distribution due to the nonthermal carriers, Δf_{NT}, at the annotated pump-probe delays. **f,** Same as **e** for the thermal carriers and the corresponding variation of Δf_T.

Furthermore, the dynamics of the two, nonthermal and thermal, electronic sub-population can be fully characterised by the temporal evolution of their energetic degrees of freedom, namely the excess energy density $N(t)$, in Supplementary Figure 14c, and the electron temperature increase $\Delta\Theta_E(t)$, in Supplementary Figure 14d (refer to Eqs. S10a-S10c, Section V). The nonthermal carriers undergo a rapid rise (N is directly coupled to the incident pump pulse, Eq. S10a) and fully relax back to equilibrium within a few hundreds of femtoseconds. This timescale is consistent with prior experimental observations[19,28,29] and theoretical descriptions of the scattering events experienced by photoexcited electrons towards equilibrium[21,25,30].

Likewise, the electronic temperature increase follows a similar dynamics, much faster than what is typically observed in plasmonic nanostructures. The origin of this peculiar behaviour is indeed the spatial diffusion across the picocavity, as illustrated in Section VI. Note that both $N(t)$ and $\Delta\Theta_E(t)$ have a non-zero value for negative pump-probe time delays. This is consistent with the fact that the ultrafast dynamics here displayed are those induced by the combination of the pump (centred around $t_d = 0$) and the probe pulses. Then, from Eqs. S8 and S9, the variations of the electron energy occupancy distributions Δf_{NT} and Δf_T associated with these nonequilibrium dynamics can be determined. We report these terms in Figs. S14e and S14f for nonthermal and thermal carriers, respectively. The nonthermal contribution is strongly dispersed in electronic energies, reaching states with energies as high as the absorbed (both pump and probe) photon energies, while thermal carriers are mostly localised around the Fermi level. Such a distinct occupancy distribution associated with the two carrier sub-populations explains the drastic difference of their relative contributions to the aS emission modulation.

Supplementary Note 8: Calculations of the picocavity LPDOS in the presence of a graphene nanoribbon (GNR)

Further numerical calculations were performed to complement the measurements conducted on the plasmonic picocavity when a graphene nanoribbon (GNR) is inserted into the sub-nm gap between the STM tip and the flat Au surface (refer to Fig. 4 in the main text). In particular, we examined the local photonic density of states (LPDOS) of the picocavity ρ_{phot} in the presence of the GNR as a function of the nanoribbon position. A simulation equivalent to the one presented in Section II was implemented, allowing us to determine the system's electromagnetic response under dipolar excitation, and to calculate ρ_{phot} (of which again we analysed specifically the radiative component) for the modified cavity configuration. The GNR was accounted for in the model by defining a rectangular 2D domain (sheet of zero thickness) of size 5 nm × 1 nm (consistent with the experimental sample) lying on the Au substrate, with an assigned conductivity $\sigma(\omega)$. The analytical expression of $\sigma(\omega)$ given in previous reports[31,32] was considered, assuming typical values for the parameters of graphene that were not characterised experimentally. Specifically, we used a Fermi energy of 0.2 eV, and a Drude scattering time of 100 fs. The contribution of the GNR to the optical response of the whole system was then expressed in terms of an induced surface current $\boldsymbol{j} = \sigma\boldsymbol{E}$, with $\boldsymbol{E}$ the electric field solution of Maxwell's equations.

To investigate the impact of the relative position of the GNR on the photonic properties of the picocavity, calculations were conducted by varying gradually the in-plane position of the GNR with respect to the Au tip. Starting from the precise centre of the cavity, right below the tip ($x_{GNR} = 0$, $y_{GNR} = 0$, as sketched in

Supplementary Figure 15a, top), the sheet was moved 2.5 nm apart in the *y*-direction (Supplementary Figure 15a, bottom), namely completely out of the cavity.

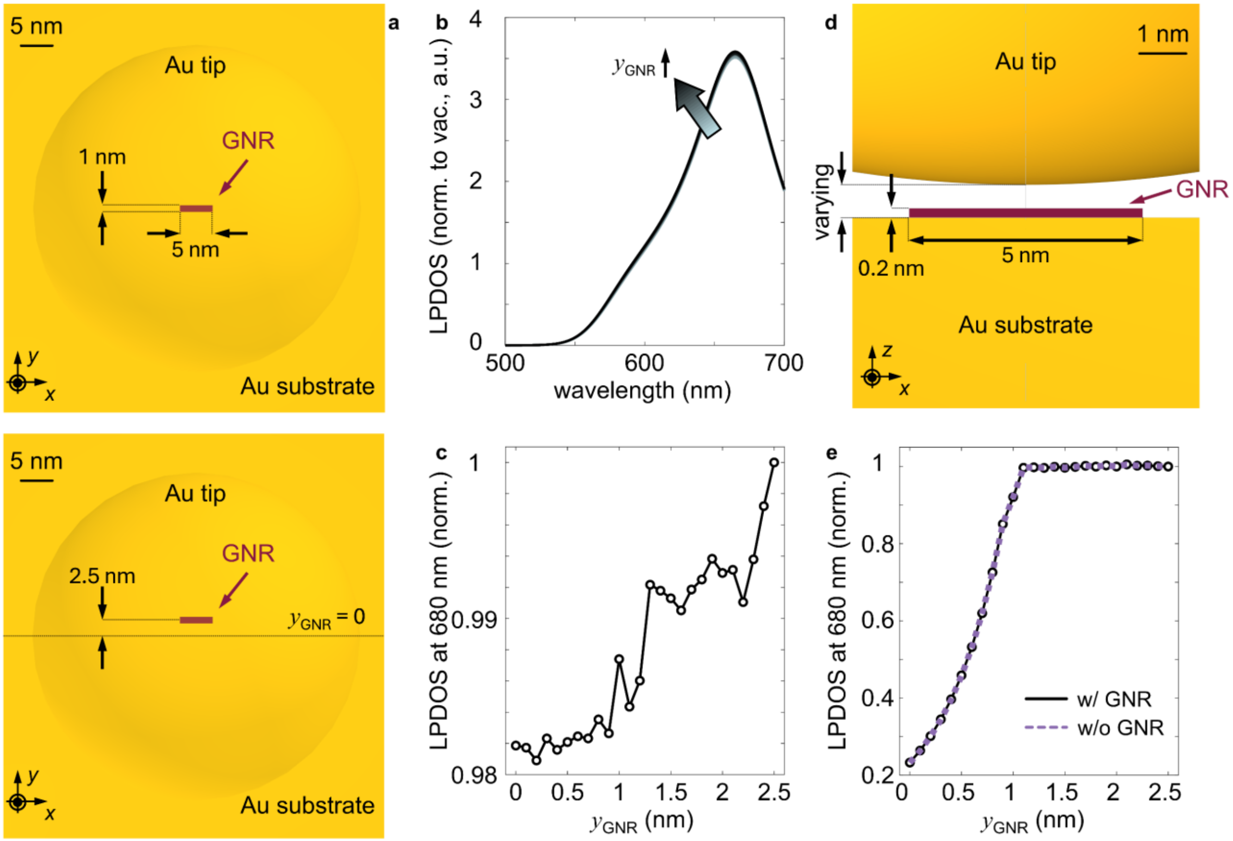

Supplementary Figure 15: LPDOS with GNR. a, Top view of the cavity, where a sheet of GNR is inserted and moved from the centre (top) outside the cavity (bottom). **b,** LPDOS calculated at various positions of the GNR. **c,** LPDOS at 680 nm as a function of the *y*-coordinate of the GNR. **d,** Schematic of the 3D GNR model, where the tip-distance changes according to the in-plane position of the GNR. **e,** LPDOS at 680 nm calculated for the 3D GNR by concurrently moving the GNR in the plane and varying the distance between the tip and the Au substrate surface. Dashed line is the calculation when the GNR is not in place. The tip-substrate distance is varied linearly with the y_{GNR} from 0.7 nm (for y_{GNR} = 0) to 0.5 nm (when y_{GNR} = 1.25 nm).

For each position of the nanoribbon, the corresponding LPDOS was computed, according to the formulas given in Section II. Despite the displacement of the graphene sheet, the calculated spectra of ρ_{phot} did not exhibit any significant change. Our main results are summarised in Supplementary Figure 15b, where indeed all curves, each of which corresponds to a distinct $y_{GNR,}$ mostly overlap. More specifically, in

Supplementary Figure 15c we examined the evolution of the LPDOS at a wavelength of 680 nm, i.e., at which the aS signal assigned to hot carriers was recorded in the experiments (refer to Fig. 4 in the main text). Again, only minor changes were observed when moving the nanoribbon across the picocavity. In these terms, our numerical model did not suggest that a photonic Au-GNR interaction could cause substantial variations in the LPDOS.

In addition to this set of simulations, we conducted some complementary calculations of the same kind, where we yet described the GNR as a 3D layer of finite thickness $t_{GNR} = 0.2$ nm, and same in-plane size (5 nm × 1 nm). The modelled geometry was modified accordingly (schematically depicted in Supplementary Figure 15d), and, in terms of graphene optical properties, we introduced[33,34] an anisotropic dielectric permittivity with in-plane components $\varepsilon_{xx}(\omega) = \varepsilon_{yy}(\omega) = 1 - i\sigma(\omega)/(\omega\varepsilon_0 t_{GNR})$. Indeed, although the optical description of GNRs characterised by a conductivity $\sigma(\omega)$ is well-established, we introduced a finite thickness of the GNR to examine possible effects resulting from the changes in the cavity effective size. Considering that: (i) the GNR thickness t_{GNR} estimated experimentally was comparable with the gap distance between the tip and the Au substrate; and (ii) the STM operated in constant current mode, thus the tip moved upward (from 0.5 nm up to ~ 0.65 nm from the Au surface) when sitting on top of the GNR, our additional simulations aimed at reproducing these spatial changes in the picocavity configuration, and assessing their impact. The calculations were therefore performed following the same procedure as the 2D-sheet case, but this time each value of y_{GNR} corresponded to a distinct value of the distance between the tip and the Au substrate, starting from 0.65 nm for $y_{GNR} = 0$, and back to 0.5 nm when $y_{GNR} = 1.25$ nm (i.e., the entire 1-nm-wide GNR can be considered to be outside the cavity). The so-obtained LPDOS, evaluated at 680 nm, is reported in Supplementary Figure 15e (solid line), and seems to exhibit a variation (by almost a factor of 4) with increasing y_{GNR}. We argue however that the observed trend should be ascribed to the change in the size of the gap between the STM tip and the Au substrate, rather than to photonic effects introduced by the GNR. To ascertain this argument, we repeated the same calculation with the moving tip, yet by removing the GNR from the picocavity. The resulting ρ_{phot}, shown as well in Supplementary Figure 15e (dashed line), follows practically the same trend as the study with the GNR in place, indicating that the gap size is in fact responsible for the variations of LPDOS in our calculations.

The measured line profiles of the variation of the anti-Stokes signal intensity when the nanotip makes a lateral line scan across the GNR as shown in Fig. 4b has peculiar features at the edges of the nanoribbon (i.e. local maxima). These feature cannot be reproduced by the simulation of the LPDOS accounting only for the dielectric property of the GNR. Thus, these features should be originating from the contributions of

the electronic structure of the GNR to the LPDOS (as also argued in the main text), which were not considered in the simulations.

Supplementary References

1 Baumberg, J. J. Picocavities: a Primer. *Nano Letters* **22**, 5859-5865 (2022). https://doi.org/10.1021/acs.nanolett.2c01695

2 Martín-Jiménez, A. *et al.* Unveiling the radiative local density of optical states of a plasmonic nanocavity by STM. *Nature Communications* **11**, 1021 (2020). https://doi.org/10.1038/s41467-020-14827-7

3 Johnson, P. B. & Christy, R. W. Optical Constants of the Noble Metals. *Physical Review B* **6**, 4370-4379 (1972). https://doi.org/10.1103/PhysRevB.6.4370

4 Etchegoin, P. G., Le Ru, E. C. & Meyer, M. An analytic model for the optical properties of gold. *The Journal of Chemical Physics* **125** (2006). https://doi.org/10.1063/1.2360270

5 Husnik, M. *et al.* Quantitative Experimental Determination of Scattering and Absorption Cross-Section Spectra of Individual Optical Metallic Nanoantennas. *Physical Review Letters* **109**, 233902 (2012). https://doi.org/10.1103/PhysRevLett.109.233902

6 Carminati, R. *et al.* Electromagnetic density of states in complex plasmonic systems. *Surface Science Reports* **70**, 1-41 (2015). https://doi.org/https://doi.org/10.1016/j.surfrep.2014.11.001

7 Novotny, L. & Hecht, B. *Principles of nano-optics*. (Cambridge university press, 2012).

8 Sanders, S. & Manjavacas, A. Analysis of the Limits of the Local Density of Photonic States near Nanostructures. *ACS Photonics* **5**, 2437-2445 (2018). https://doi.org/10.1021/acsphotonics.8b00225

9 Cao, S. *et al.* Probing the Radiative Electromagnetic Local Density of States in Nanostructures with a Scanning Tunneling Microscope. *ACS Photonics* **7**, 1280-1289 (2020). https://doi.org/10.1021/acsphotonics.0c00264

10 Bohren, C. F. & Huffman, D. R. *Absorption and scattering of light by small particles / Craig F. Bohren, Donald R. Huffman*. (Wiley, 2004).

11 Jollans, T., Caldarola, M., Sivan, Y. & Orrit, M. Effective Electron Temperature Measurement Using Time-Resolved Anti-Stokes Photoluminescence. *The Journal of Physical Chemistry A* **124**, 6968-6976 (2020). https://doi.org/10.1021/acs.jpca.0c06671

12 Sivan, Y. & Dubi, Y. Theory of "Hot" Photoluminescence from Drude Metals. *ACS Nano* **15**, 8724-8732 (2021).

13 Sivan, Y. *et al.* Crossover from Nonthermal to Thermal Photoluminescence from Metals Excited by Ultrashort Light Pulses. *ACS Nano* **17**, 11439-11453 (2023). https://doi.org/10.1021/acsnano.3c01016

14 Lin, Z., Zhigilei, L. V. & Celli, V. Electron-phonon coupling and electron heat capacity of metals under conditions of strong electron-phonon nonequilibrium. *Physical Review B* **77**, 075133 (2008). https://doi.org/10.1103/PhysRevB.77.075133

15 Rosei, R., Antonangeli, F. & Grassano, U. M. d bands position and width in gold from very low temperature thermomodulation measurements. *Surface Science* **37**, 689-699 (1973). https://doi.org/https://doi.org/10.1016/0039-6028(73)90359-2

16 Sun, C. K., Vallee, F., Acioli, L. H., Ippen, E. P. & Fujimoto, J. G. Femtosecond-Tunable Measurement of Electron Thermalization in Gold. *Phys. Rev. B* **50**, 15337-15348 (1994).

17 Schirato, A., Maiuri, M., Cerullo, G. & Valle, G. D. Ultrafast hot electron dynamics in plasmonic nanostructures: experiments, modelling, design. *Nanophotonics* **12**, 1-28 (2023). https://doi.org/doi:10.1515/nanoph-2022-0592

18 Carpene, E. Ultrafast laser irradiation of metals: Beyond the two-temperature model. *Phys. Rev. B* **74** (2006). https://doi.org/https://doi.org/10.1103/PhysRevB.74.024301

19 Della Valle, G., Conforti, M., Longhi, S., Cerullo, G. & Brida, D. Real-time optical mapping of the dynamics of nonthermal electrons in thin gold films. *Phys. Rev. B* **86** (2012). https://doi.org/https://doi.org/10.1103/PhysRevB.86.155139

20 Besteiro, L. V. *et al.* The fast and the furious: Ultrafast hot electrons in plasmonic metastructures. Size and structure matter. *Nano Today* **27**, 120-145 (2019). https://doi.org/https://doi.org/10.1016/j.nantod.2019.05.006

21 Liu, J. G., Zhang, H., Link, S. & Nordlander, P. Relaxation of Plasmon-Induced Hot Carriers. *Acs Photonics* **5**, 2584-2595 (2018). https://doi.org/DOI: 10.1021/acsphotonics.7b00881

22 Schirato, A. *et al.* Quantifying Ultrafast Energy Transfer from Plasmonic Hot Carriers for Pulsed Photocatalysis on Nanostructures. *ACS Nano* **18**, 18933-18947 (2024). https://doi.org/https://doi.org/10.1021/acsnano.4c01802

23 Schirato, A. *et al.* Transient optical symmetry breaking for ultrafast broadband dichroism in plasmonic metasurfaces. *Nature Photonics* **14**, 723-727 (2020). https://doi.org/10.1038/s41566-020-00702-w

24 Schirato, A., Crotti, G., Proietti Zaccaria, R., Alabastri, A. & Della Valle, G. Hot carrier spatio-temporal inhomogeneities in ultrafast nanophotonics. *New Journal of Physics* **24**, 045001 (2022). https://doi.org/10.1088/1367-2630/ac6009

25 Chang, L. *et al.* Electronic Structure of the Plasmons in Metal Nanocrystals: Fundamental Limitations for the Energy Efficiency of Hot Electron Generation. *ACS Energy Lett.* **4**, 2552-2568 (2019). https://doi.org/https://doi.org/10.1021/acsenergylett.9b01617

26 Schirato, A., Maiuri, M., Cerullo, G. & Della Valle, G. Ultrafast hot electron dynamics in plasmonic nanostructures: experiments, modelling, design. *Nanophotonics-Berlin* **12**, 1-28 (2023). https://doi.org/https://doi.org/10.1515/nanoph-2022-0592

27 Sivan, Y. & Spector, M. Ultrafast Dynamics of Optically Induced Heat Gratings in Metals. *ACS Photonics* **7**, 1271-1279 (2020). https://doi.org/10.1021/acsphotonics.0c00224

28 Heilpern, T. *et al.* Determination of hot carrier energy distributions from inversion of ultrafast pump-probe reflectivity measurements. *Nat. Commun.* **9**, 1853 (2018). https://doi.org/https://doi.org/10.1038/s41467-018-04289-3

29 Brown, A. M. *et al.* Experimental and Ultrafast Carrier Dynamics in Plasmonic Nanoparticles. *Phys. Rev. Lett.* **118** (2017). https://doi.org/https://doi.org/10.1103/PhysRevLett.118.087401

30 Bernardi, M., Mustafa, J., Neaton, J. B. & Louie, S. G. Theory and computation of hot carriers generated by surface plasmon polaritons in noble metals. *Nat. Commun.* **6**, 7044 (2015). https://doi.org/https://doi.org/10.1038/ncomms8044

31 Falkovsky, L. A. Optical properties of graphene. *Journal of Physics: Conference Series* **129**, 012004 (2008). https://doi.org/10.1088/1742-6596/129/1/012004

32 Koppens, F. H. L., Chang, D. E. & García de Abajo, F. J. Graphene Plasmonics: A Platform for Strong Light–Matter Interactions. *Nano Letters* **11**, 3370-3377 (2011). https://doi.org/10.1021/nl201771h

33 Majérus, B., Dremetsika, E., Lobet, M., Henrard, L. & Kockaert, P. Electrodynamics of two-dimensional materials: Role of anisotropy. *Physical Review B* **98**, 125419 (2018). https://doi.org/10.1103/PhysRevB.98.125419

34 Emani, N. K., Kildishev, A. V., Shalaev, V. M. & Boltasseva, A. Graphene: A Dynamic Platform for Electrical Control of Plasmonic Resonance. *Nanophotonics* **4**, 214-223 (2015). https://doi.org/doi:10.1515/nanoph-2015-0014
